# Analysing the Influence of Health Insurance Status on Peoples' Health Seeking Behaviour in Rural Ghana

**DOI:** 10.1155/2017/8486451

**Published:** 2017-05-08

**Authors:** Benedict Osei Asibey, Seth Agyemang

**Affiliations:** Department of Geography and Rural Development, Faculty of Humanities and Social Sciences, Kwame Nkrumah University of Science and Technology, University Post Office, Private Mail Bag, Kumasi, Ghana

## Abstract

This paper examines the influence of health insurance status on healthcare use in rural Ghana using 286 sampled respondents from four rural communities in the Bekwai Municipality. Data were obtained using structured interview and Pearson's Chi square and bivariate regressions were used to analyse data. The results show low healthcare utilization among study participants, with most respondents having irregular use (43.5%) or rare use (43.3%). Respondents with health insurance utilized healthcare more than those without health insurance, the results being statistically significant (df = 4; *n* = 283, *p* = 0.000). The bivariate analysis revealed that health insurance status has a positive and significant influence on utilization (*β* = 1.284; *p* value = 0.000). The study recommends promotion and improvement of services of the National Health Insurance Scheme as effective strategy to improve healthcare consumption by the rural people. The expansion of health insurance services to all sections of the population is also recommended.

## 1. Introduction

Good health is a major contributor to quality of life, enabling people to fully participate in productive activities for wealth creation. It is also a key determinant of human development [1]. The World Health Organization (WHO) recognizes health as a priority in its own right and a central input in development and poverty reduction [2]. Healthcare utilization thus comes up as significant public health and policy issue and a matter of concern to stakeholders, reflecting efforts to both improve health outcomes and meet international obligations to make health care accessible.

The extent of healthcare use is however related to several factors including (physical) access, cost of service, and a host of other socioeconomic and personal characteristics [3]. In Sub-Saharan Africa the economic factors of income and service cost strongly influence health seeking behaviour, especially for the rural and low income folks [4]. The need to improve access and use of healthcare by minimizing the socioeconomic barriers of service cost and low incomes comes to the fore, paving the way for a health financing approach that shares risks across population groups over time, and replaces out-of-pocket health expenditure.

Increasing global attention is being drawn towards universal health coverage, and policies on health insurance play a strategic role in that drive. As a form of prepaid financing system, health insurance makes possible collective pooling of risks and the redistribution of financial resources in such a way that assures financial protection against the cost of illness [5–7]. Access to health insurance thus comes up a game changer to significantly improve utilization of healthcare by providing financial protection against disastrous out-of-pocket healthcare expenditures. The literature establishes direct linkages between health insurance coverage and cost and actual usage of healthcare [6–9]. However, health insurance as a health financing option is poorly implemented in Sub-Saharan Africa [6, 10], and Ghana's recent “moderately successful” experience is held up as a model not only for the region, but also on the global arena. Factors such as low enrolment [6, 11, 12] and high rates of dropout [13] help to explain the slow-paced expansion of insurance schemes in Sub-Saharan Africa including Ghana. It is also observed that health insurance enrolment in most developing countries tends to be unbalanced as schemes favour the rich few as against the poor and vulnerable majority most of whom are found in rural areas [14]. Low scheme coverage nevertheless imposes calamitous health spending patterns associated with absence of insurance cover.

Ghana, a lower middle income country with even more ambitious development targets, requires a healthy population as a key ingredient for sustained economic and social progress, hence a clear policy focus on health. Addressing financing is to touch on a key component of the healthcare challenges in Ghana. Before independence in 1957, healthcare in Ghana was mainly financed by out-of-pocket payments at point of service use [15]. This changed from a practically free system under the communally inclined First Republic, and then back to a system of payment for health services, culminating in the dreaded “Cash and Carry” system where patients were made to pay for their drugs and other health consumables [15, 16]. This was in operation until 2004 when the National Health Insurance Scheme (NHIS) was born, established under Act 650 of 2003 with the objective of making available basic healthcare services to individuals living in the country by way of private and mutual health insurance schemes.

A healthy rural population is needed in Ghana for several reasons. First, the rural sector contributes immensely to the growth and development of the country in several ways such as agriculture to produce food for local consumption as well as for exports for foreign exchange, which forms a bigger proportion of the national income. The rural sector also produces raw materials to feed local manufacturing industries. Also, a large percentage of the Ghanaian population lives in rural areas. The 2010 Ghana Population and Housing Census revealed that almost half (49.1%) of Ghanaians live in rural areas, and, in the Bekwai Municipality where this study was undertaken, out of total population of 118,024, about 97,277 representing 82.4% reside in rural areas [17], implying that rural people form a significant portion of Ghana's workforce.

Nevertheless, when it comes to health, rural areas in Ghana face difficulties with transport and communication, as well as challenges of shortages of doctors, nurses, and other health professionals and health service providers. The rural people in Ghana are also mostly low income people, which imply that they are least able to cater for the cost of accessing health services when the need arises. These challenges lead to low levels of healthcare utilization even in the face of ill health, with several negative consequences [3, 18]. According to the Ghana Ministry of Health [19], though efforts are being made to improve health in the country, actual utilization of healthcare still remains a significant problem among the rural population. However, achieving a decent health status among the populace and realizing both national and international goals requires that access and use of quality healthcare is improved significantly in the countryside [20]. This has however been terrible in Ghana [19]. Poor health conditions of the rural population will therefore have several negative consequences on growth and development. First, the rural people's contribution to development is affected which implies a significant loss. Agricultural production for both local consumption and exports will be reduced [20]. Also, the country's labour force will be reduced. Therefore, efforts by the government and development partners to improve the health conditions of the Ghanaian population should target the rural people in order to fully achieve its goals.

However, one way of improving access and use of healthcare by the rural people is to reduce or eliminate barriers of financing which includes the elimination of out-of-pocket expenditure, and this calls for health insurance. Though a subject of much research in the literature not much is known about the influence of health insurance status on the use of healthcare by rural people. This study thus examines the influence of health insurance status on healthcare use in rural Ghana, with selected rural communities in the Bekwai Municipal as case study. The Bekwai Municipality where the study was conducted has as many as 82.4% of the population residing in rural areas [17]. However, the consumption of healthcare in Ghana is generally low but the level in rural areas is worse [19], due to several identified factors including unbalanced distribution of health facilities between the rural and urban areas [21, 22], low incomes, and high service cost [22]. This study investigates the influence of health insurance status on rural people's healthcare utilization. The research objectives are thus to determine the insurance status of rural people in the municipality; assess the use of healthcare by rural people; and examine the influence of insurance status on use of healthcare.

The study is guided by the following hypothesis:

1. H_0_: There is no significant difference in the use of healthcare between insured and uninsured people.
2. H_0_: Health insurance has no significant influence on the use of healthcare.

## 2. Data and Methods

### 2.1. Study Design and Sampling

This study formed part of a larger research that investigated the determinants of healthcare use in rural Ghana within the Bekwai Municipality. The study adopted a population-based cross-sectional and quantitative survey design to investigate the influence of insurance status on the use of healthcare, using four (4) selected communities, namely, Senfi, Sehwi, Chiransah, and Huntado. The Bekwai Municipality was selected because of its rural character with its very few health facilities. These communities were selected because of their nearness to health facilities. Systematic random sampling was used to select the housing units from which one household representative was randomly chosen as a respondent.

A total of 286 respondents were selected from a combined 1,003 housing units in the four communities, with the selection based on the total number of housing units in each community. The respondents constituted only heads of households. However, in a situation where the head of a selected household is not available, any member of the household aged 18 years and above and willing to participate was selected. A list of housing units was collected for each community from the Municipal Assembly and was used as sample frame. The total numbers of housing units in each of the study communities were 373, 422, 149, and 68 for Senfi, Huntado, Sehwi, and Chiransah, respectively whereas the samples selected from each community were 103, 116, 41, and 26 for Senfi, Huntado, Sehwi, and Chiransah, respectively. A sampling interval of 4 was used to select the housing units in Senfi and Huntado. This sampling interval was calculated by dividing the total number of households in Senfi and Huntado (373 and 422, resp.) by the sample sizes of 103 and 116 to be, respectively, selected from the two communities. The sample interval of 3 for selection of housing units in Sehwi and Chiransah was also derived by dividing the total number of housing units of 149 and 68, respectively, by the sample sizes of 41 and 26. In every community, one house was randomly selected between the first house on the list and the sample interval and the sample selection continued using the sample interval for every community. In a situation where there were more than one household in a dwelling, only one household was randomly selected with the assumption that all the households had similar characteristics. Also, in a situation where nobody was found in a selected house or where no member of a particular house was willing to participate, the next house was selected and the same sample interval was used to select the subsequent houses from there.

### 2.2. Data Collection

Primary data was collected using structured questionnaire. The low literacy levels in the study communities necessitated adoption of the interviewer-administered approach. The interactions were also done in the local Twi language which is the most patronized dialect in the study area. Participation in the study was voluntary, and informed consent was obtained before interviewing began. All the respondents were adults aged 18 years and above. A total of 283 out of the 286 rural people sampled responded to the questionnaire giving a response rate of 99%.

### 2.3. Variables and Their Operationalisation

The dependent variable for the study was healthcare use, and this was operationalised as the number of times a respondent visited a health facility in the last four (4) times of illness spells. Healthcare use comprised total doctor visits, general practitioner visits, and specialist visits and was ranked following Buor [21] from “0” for nonattendance of health centre and described as “rarely” to “4” for attending for all four and described as “very regular.” The questionnaire included both continuous and ranked items. Insurance status and employment status were entered as dichotomous variables. Insurance status was defined as insured = 1 and uninsured = 0, and employment status was defined as employed = 1 and unemployed = 0. Insured respondents were defined as individuals with valid National Health Insurance cards and entitled to free medical care within the past 12 months preceding the survey. Health status was measured using self-assessed criteria, rated on a scale from 1 to 5 and described as “very bad” to “very good,” respectively. Quality of service was rated on a scale from 1 to 5 and described from very poor to very good, respectively. Variables such as age, years of education, and household size were entered as continuous variables.

### 2.4. Statistical Analyses

Continuous and ranked data were used in the quantitative analysis and bivariate regression was run to determine the effect of insurance status on use of healthcare. A 0.05 or less probability (*p* ≤ 0.05) was considered significant. The confidence in the bivariate regression data was determined using the adjusted coefficient of determination (adjusted *R*^2^). Before the regression analysis, descriptive statistics were conducted to describe the background characteristics of the respondents. Also, Pearson's Chi square (*χ*^2^) tests were done to compare the demographic and background characteristics of respondents and their healthcare use in terms of insurance status. All data analyses were done using the Statistical Product for Service Solution (SPSS) version 20.

## 3. Results and Discussion

### 3.1. Characteristics of the Sample

Table 1 shows respondents' background characteristics by their health insurance status. Of the respondents, 53.5% had no health insurance coverage. In total there were more females (55.5%) compared to males (44.5%). Some 38% had no formal education while 36% had been educated up to the basic level. However, a significant 17.1% had had tertiary education.

Older people were more uninsured in terms of percentage though the difference was not significant (*p* > 0.050). Majority (82.3%) of the respondents were employed. Farming was the major occupation (46.8%), followed by artisanship (27.7%). Other livelihood activities included trading and public/civil service. The employed (75.2%) were more insured than the unemployed (24.8%), and the difference was significant (*p* < 0.050). Majority (72.4%) of the respondents were married, and these were more likely to be registered than the unmarried (74.4% as against 25.6%), at a significant difference of *p* < 0.050. Majority (72.4%) of the respondents came from homes with household size of more than five people, and there was a significant difference in health insurance status for household size (*p* < 0.010). Monthly income for respondents was also generally low: 43.8% had incomes of GH₵ 100 and below while 36.7% had between GH₵ 101 and GH₵400. There was however a significant difference among the income categories in terms of insurance status (*p* < 0.02).

### 3.2. Use of Healthcare by Respondents

Results on the extent of healthcare use by respondents are presented in Table 2. The survey reveals generally low use of healthcare service, since only a few people attended health facilities for two or more times in their last four illnesses preceding the survey. Most respondents visited health facilities either irregularly (43.5%) or rarely (43.3%). Respondents with active insurance cover utilized healthcare more than those without active insurance cover.

The bivariate analysis to compare the extent of healthcare use between the insured and uninsured respondents also resulted in a statistically significant difference (df = 4; *n* = 283, *p* = 0.000). This means that there was a significant difference in healthcare use between insured and uninsured participants. This may be due to weaker perceived health status by the insured participants hence their higher perceived need for healthcare. Insured respondents significantly perceived quality of healthcare provided and attitude of staff to be better than uninsured.

### 3.3. Influence of Health Insurance Status on the Use of Health Care

Table 3 presents the results of the bivariate regression analysis of the influence of health insurance status (independent variable) on participants' extent of health care use (dependent variable).

The result from the bivariate regression analysis shows that health insurance status has a significant influence on use of healthcare (*p* value < 0.050). The beta coefficient shows that respondents with active health insurance status are likely to attend a health facility 1.284 times more than uninsured respondents in their last four illness spells, other things being equal. The coefficient of determination (*R*^2^) means that 34.8% of variations in respondents' healthcare use are accounted for by their insurance status, leaving the other proportion to be accounted for by other factors.

## 4. Discussion

An examination of the influence of health insurance status on use of healthcare services in rural Ghana was the focus of this study, using data gathered from the Bekwai Municipality. Specifically the study highlighted the differences between insured and uninsured people in terms of health seeking behaviour.

Use of healthcare by the rural folks was generally found to be low, and there was a significant difference between the insured and the uninsured. The study found that respondents with active health insurance cover generally tended to use healthcare more frequently than those with no active insurance cover. In other words, respondents who had actively registered under health insurance schemes tended to visit health facilities more frequently than those who had not. This result is worrying since low levels of healthcare services use has negative effects on the health conditions of the population, which is very vital for the socioeconomic development of the municipality and the nation as a whole. Reasons for the insured using healthcare services more than the uninsured may include weaker perceived health status by the former which makes them have a higher perceived need for healthcare. Another reason might be that the cost of accessing healthcare tends to be cheaper for the insured more than those not insured. Actively insured people find access and use of healthcare cheaper than the uninsured who have to pay for the use of healthcare services, and this prevents them from visiting health facilities when the need arises. Also, the insured were mostly found to be highly educated making them more likely to take advantage of health care services [23, 24] since they have greater awareness of the existence and value of preventive as well as curative health care services. Appropriate government policies are required to improve the rate of use for the rural people by way of expanding health insurance services to all. Promoting and improving the services of the National Health Insurance Scheme is a very effective strategy to improve healthcare consumption by the rural people. This finding supports similar studies in Ahafo Ano South District in Ghana [21], slum areas in Accra [6, 7, 9, 25], and South Korea [26, 27] that people with health insurance cover utilize healthcare services more than those with no health insurance cover.

The study also found from the bivariate regression analysis that health insurance status has a positive and significant influence on use of healthcare services in the Bekwai Municipality. A number of factors may account for this finding. First, health insurance has a positive influence on the use of healthcare by way of making healthcare more accessible to the people in terms of reduced cost of service. Registered people no longer have to pay for healthcare services such as consulting, laboratory tests, drugs, and other health consumables. Also, health insurance reduces the financial burden of people since they no longer have to spend huge portion of their incomes on healthcare but rely on the insurance services. This finding implies that people with active health insurance cover are more likely to access and use healthcare services than those without active insurance cover when the need arises. Health insurance services promote the use of healthcare services for the rural dwellers leading to positive health outcomes, and this means that it is a policy that should be expanded to all sections of the population in order to promote good health which is very significant for development. This finding of a significant influence of people's health insurance status on their use of healthcare services supports the findings of earlier studies in Israel [28, 29], Japan [30], and Ghana [31] that health insurance has a positive and significant effect on utilization of health care services as measured by admissions, inpatient days, and outpatient visits to hospitals.

Both null hypotheses for the study have been rejected. Firstly, the hypothesis that there is no significant difference in the use of healthcare between insured and uninsured people is rejected. The results revealed that people with active insurance cover utilize healthcare services more than those without insurance cover. The second hypothesis that health insurance has no significant influence on people's use of healthcare is similarly rejected. The result revealed a positive and significant influence of health insurance status on use of healthcare services, such that the insured are more likely than the uninsured to use healthcare services when the need arises.

There were some limitations with regard to the methodology. There may be a possibility of recall bias in self-reported use of healthcare. There may also be some biases during the sampling procedure. For example, study region, district, and the rural communities were all selected purposively making it statistically unrepresentative. Also, the systematic random sampling technique used in selecting the households gives the tendency of losing some vital information from the target population that was skipped over. However, the research made use of a homogeneous sampling frame and could therefore report similar cases.

## 5. Conclusions

The study has shown that healthcare use among rural folks in the Bekwai Municipality is generally low and there is therefore the need to improve access and use of healthcare services. It has shown that rural people are mostly not registered under any health insurance scheme. In view of this, all interventions geared towards improving access to insurance services should be put in place by stakeholders such as the Ghana Health Service, Ministry of Health, National Health Insurance Authority, and other NGOs.

The study has also shown a significant difference between insured and uninsured participants with regard to healthcare services use, as well as the significant and positive influence of insurance status on rate of healthcare use. Therefore, given the importance of health insurance services in improvement of healthcare use and health outcomes, interventions should be made towards expanding its coverage to everyone particularly the low income people as well as those in the rural areas. The scheme's management should highlight the communal nature of the scheme, especially targeting the relatively younger populations as well as well-informed educated individuals so as to discourage the notion that there are no incentives to enroll in health insurance schemes unless there is illness.

This study has successfully examined the essential difference in the use of healthcare services by people's insurance status. The survey has used the bivariate regression technique to demonstrate the influence of health insurance status on the use of healthcare among rural dwellers.

## Supplementary Material

The informed consent form which is the supplementary material is the document that all participants in the survey were made to sign before participating in the survey. The informed consent form provided information about the study that was conducted and it contained the several information such as Description of the research and the participation of the selected respondents, including the description of the research procedure, Location and time of the study, Risks and discomforts as well as potential benefits, Protection and confidentiality, voluntary participation, contact information of the researcher and lastly the participants' consent.

## Figures and Tables

**Table 1:** Background characteristics of study participants by health insurance status.

Variables	Categories	National Health Insurance Status				Total (*N* = 283)		*χ* ^2^ (*p* value)
		Insured (*N* = 133)		Uninsured (*N* = 150)				
		*N*	(%)	*N*	(%)	*N*	(%)	
Gender	Male	60	(45.1)	66	(44.0)	126	(44.5)	
	Female	73	(54.9)	84	(56.0)	157	(55.5)	0.473

Age	18–39	21	(15.8)	63	(42.0)	84	(29.7)	
	40–59	49	(36.8)	54	(36.0)	103	(36.4)	0.052
	60 and above	63	(47.4)	33	(22.0)	96	(33.9)	

Education	No education	49	(36.8)	58	(38.7)	107	(37.8)	
	Basic education	42	(31.6)	80	(53.3)	102	(36.0)	
	Secondary	14	(10.5)	12	(8.0)	26	(9.1)	0.000
	Tertiary education	28	(21.1)	0	(0.0)	28	(17.1)	

Employment status	Unemployed	31	(24.8)	19	(12.7)	50	(17.7)	
	Employed	100	(75.2)	131	(87.3)	231	(82.3)	0.012

Nature of occupation	Farming	38	(38.0)	70	(53.4)	108	(46.8)	
	Trading	16	(16.0)	23	(17.6)	39	(16.9)	
	Artisan	25	(25.0)	39	(29.0)	64	(27.7)	0.061
	Public/civil service	21	(21.0)	0	(0.0)	21	(8.6)	

Marital status	Single	34	(25.6)	44	(29.3)	78	(27.6)	0.020
	Married	99	(74.4)	106	(70.7)	205	(72.4)	

Household size	1–5	63	(47.4)	15	(10.0)	78	(27.6)	
	6–10	65	(48.7)	121	(80.7)	186	(65.7)	0.000
	>10	5	(3.6)	14	(9.3)	19	(6.7)	

Average monthly income (GH₵)	≤100	42	(31.6)	82	(54.7)	124	(43.8)	
	101–400	57	(42.9)	47	(31.3)	104	(36.7)	
	401–600	21	(15.8)	11	(7.3)	32	(11.2)	0.012
	601–1000	9	(6.8)	6	(4.0)	15	(5.3)	
	>1000	4	(2.9)	4	(2.7)	8	(3.0)	

**Table 2:** Selected questions regarding participants' healthcare use.

Variables	Categories	Health Insurance Status				Total		*χ* ^2^ sig (*p* value)
		Insured (*N* = 133)		Uninsured (*N* = 150)				
		*N*	(%)	*N*	(%)	*N*	(%)	
Quality of service	Very poor	0	(0.0)	7	(4.7)	7	(2.5)	
	Poor	12	(9.0)	16	(10.7)	28	(9.9)	
	Satisfactory	57	(42.7)	71	(47.3)	128	(45.2)	
	Good	51	(38.2)	15	(10.0)	66	(23.3)	**0.000**
	Very good	10	(7.5)	0	(0.0)	10	(3.6)	
	*Missing*	3	(2.6)	41	(27.3)	44	(15.5)	

Attitude of staff	Very poor	4	(3.0)	8	(5.3)	12	(4.2)	
	Poor	28	(21.1)	52	(34.7)	80	(28.3)	
	Satisfactory	58	(43.5)	53	(35.3)	111	(39.2)	
	Good	25	(18.5)	3	(2.0)	28	(9.9)	**0.001**
	Very good	15	(11.3)	0	(0.0)	15	(5.3)	
	Missing	3	(2.6)	34	(22.7)	37	(13.1)	

Healthcare use	0 (rarely)	10	(7.5)	78	(52.0)	88	(43.3)	
	1 (irregularly)	56	(42.2)	67	(44.7)	123	(43.5)	0.000
	2 (moderately)	32	(24.1)	5	(3.3)	37	(13.1)	
	3 (regularly)	21	(15.7)	0	(0.0)	21	(7.4)	
	4 (very regularly)	14	(10.5)	0	(0.0)	14	(6.9)	

**Table 3:** Bivariate regression factor for influence of insurance status on use of healthcare.

Variable	Constant	Beta coefficient	Sig (*p* value)	*R* ^2^
Insurance status	0.513	1.284	0.000	0.348

(a) Dependent variable: healthcare use.
