# Supplementary material for: Analysing the Influence of Health Insurance Status on Peoples' Health Seeking Behaviour in Rural Ghana
Source: J Trop Med. 2017 May 8;2017:8486451. doi: 10.1155/2017/8486451 (PMC5439069; doi:10.1155/2017/8486451)
Supplement: Supplementary file 1 — The informed consent form which is the supplementary material is the document that all participants in the survey were made to sign before participating in the survey. The informed consent form provided information about the study that was conducted and it contained the several information such as Description of the research and the participation of the selected respondents, including the description of the research procedure, Location and time of the study, Risks and discomforts as well as potential benefits, Protection and confidentiality, voluntary participation, contact information of the researcher and lastly the participants' consent. [file 8486451.f1.pdf]

Consent Form for Participation in a Research Study
Kwame Nkrumah University of Science and Technology

Title of Study: Determinants of Healthcare Utilization in Rural Communities in Bekwai Municipality, Ghana

Description of the research and your participation

You are invited to participate in a research study conducted by **BENEDICT OSEI ASIBEY** of the Department of Geography and Rural Development, Kwame Nkrumah University of Science and Technology. Kumasi, Ghana. The purpose of this study is to examine the determinants of health seeking behaviour in rural Ghana, with the Bekwai municipality as case study.

You are being invited to participate in this study because you are the head of this sampled household. If you agree to participate in the study, you will be required to answer various questions about your socio-demographic background, your health status and your health-seeking behaviour. Alternatively, you could be given the questionnaire containing the same items, for you to respond yourself, if you so desire.

Study Location
This will take place at your home.

Study Time
This exercise will take not more than 45 minutes.

Risks and discomforts

There are no risks, whether known or hidden, associated with participation in this study. And the information you provide will not be linked to you in any way.

Potential benefits
While no immediate direct benefits resulting from your participation in the study can be assured, it is expected that the results of the study will guide stakeholders to put in place measures that address barriers to access and use of healthcare in rural Ghana.

Protection of confidentiality

Your privacy and anonymity will be protected both during and after the research. Your identity will not be revealed in any way, and the blind nature of the research protocol will ensure that no information you provide can be linked to you in any way. This applies to both the tabulated data as well as any publication that will result from this study.

Voluntary participation

Your participation in this research study is strictly voluntary. You are not under any duress or obligation to participate, and you can withdraw your consent to participate at any time. Also, you may refuse to respond to any question in the questionnaire you do not want to answer. You will not be penalized in any way should you decide to withdraw from this study or opt not answer any question.

Contact information

If you have any other concerns about this study, please contact **BENEDICT OSEI ASIBEY** at The Department of Geography and Rural Development, Kwame Nkrumah University of Science and Technology or you can call me on 0268963101.

Consent

I, having read and understood the terms and conditions associated with the above exercise, do hereby

1. Agree to participate ☐
2. Refuse to participate ☐

If you agree to participate in this study, please sign and date below.

_______________________________ _____________

Participant's Name Date

_______________________________ _____________

Participant's Signature Date

_______________________________ _____________

Person Obtaining Consent Date
